# Two-dimensional measurements of receptor-ligand interactions

**DOI:** 10.3389/fmolb.2023.1154074

**Published:** 2023-02-17

**Authors:** Songjie Zheng, Min Zou, Yingfeng Shao, Huaping Wu, Helong Wu, Xiaohuan Wang

**Affiliations:** ^1^ Institute of Mechanics, Chinese Academy of Sciences, Beijing, China; ^2^ School of Engineering Science, University of Chinese Academy of Sciences, Beijing, China; ^3^ College of Mechanical Engineering, Zhejiang University of Technology, Hangzhou, China; ^4^ Department of Rehabilitation Medicine, Peking University Third Hospital, Beijing, China

**Keywords:** receptor-ligand interaction, binding kinetics, kinetic on-rate, kinetic off-rate, binding affinity, two-dimensional measuring methods

## Abstract

Gaining insight into the two-dimensional receptor-ligand interactions, which play a significant role in various pivotal biological processes such as immune response and cancer metastasis, will deepen our understanding of numerous physiological and pathological mechanisms and contribute to biomedical applications and drug design. A central issue involved is how to measure the *in situ* receptor-ligand binding kinetics. Here, we review several representative mechanical-based and fluorescence-based methods, and briefly discuss the strengths and weaknesses for each method. In addition, we emphasize the great importance of the combination of experimental and computational methods in studying the receptor-ligand interactions, and further studies should focus on the synergistic development of experimental and computational methods.

## 1 Introduction

**FIGURE 1:**
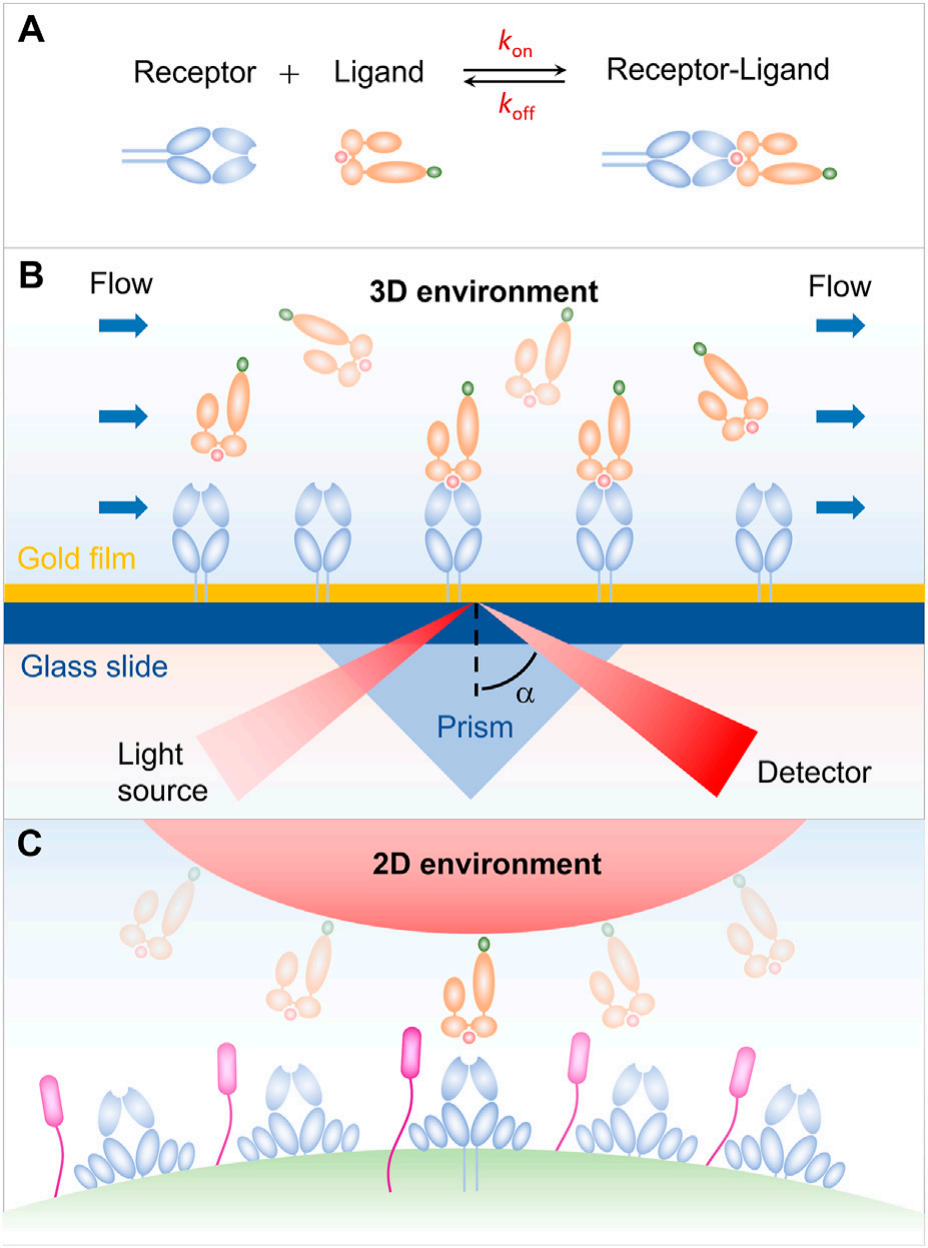
Receptor-ligand interaction in three-dimensional (3D) and two-dimensional (2D) environments. **(A)** Schematic of an affinity reaction. **(B)** Interaction in 3D environment. **(C)** Interaction in 2D environment.

The interactions between receptors and ligands in cell adhesion are fundamental for cell communication (Hoffmann and Slansky, 2020; Overall et al., 2020; Li et al., 2021a; Liu et al., 2021; Zareie et al., 2021; Ng Chau et al., 2022; Szeto et al., 2022) and play a significant role in various pivotal biological processes such as signal transduction, immune response, tissue development, and cancer metastasis (Su et al., 2016; Wang et al., 2019; Xiong et al., 2020; Li et al., 2021b; Li et al., 2021c; Li et al., 2021e; Giampazolias et al., 2021). Studying the receptor-ligand interactions will deepen our understanding of cellular physiological and pathological mechanisms (Dustin et al., 2001; Czöndör et al., 2013; Lee et al., 2013; Li et al., 2018) and contribute to biomedical applications and drug design (Dinamarca et al., 2012; Sindi and Dodd, 2015; Kim et al., 2017; Logtenberg et al., 2020; Zhang et al., 2020). The receptor-ligand interactions can be generally characterized by their binding kinetics that involves kinetic on-rate *k*_on_, kinetic off-rate *k*_off_ and binding affinity *K* = *k*_on_/*k*_off_ (Figure 1A). The on-rate *k*_on_ and off-rate *k*_off_ describe the velocity of receptor-ligand complex formation and dissociation, respectively. The binding affinity *K* quantifies the receptor-ligand binding strength (Li et al., 2017a; Li et al., 2017b; Overall et al., 2020; Li et al., 2023). Establishing the relationship between receptor-ligand binding kinetics and cellular responses is bound to help pharmaceutical development greatly. A central issue involved is how to measure the receptor-ligand binding kinetics.

Our early understanding on the receptor-ligand binding kinetics is mainly from the surface plasmon resonance (SPR) measurement for the soluble receptors and ligands in solution. As shown in Figure 1B, either receptors or ligands are typically immobilized on a sensor chip in the SPR experiments, and then flowing the binding partner over the sensor chip. The receptor-ligand binding leads to a change in the mass of the chip surface layer, which in turn shifts the angle for total internal reflection. The kinetic information of receptor-ligand interaction, i.e., *k*_on_, *k*_off_, and *K*, is then derived by analyzing the SPR angle shift over time in combination with thermodynamic theories (Schuck, 1997; Rich and Myszka, 2000; McDonnell, 2001; Li et al., 2019). SPR experiments have provided us with important and enlightening insight into the receptor-ligand binding kinetics. Note that, the SPR measurements are performed in three-dimensional (3D) environment, whereas the *in vivo* interaction of receptors and ligands occurs in two dimension (2D) since receptors and ligands are anchored in two apposing cell membrane or a cell membrane and a matrix (Figure 1C), leading to the difference in dimension for binding affinity *K* (L^3^ in 3D and L^2^ in 2D) and kinetic on-rate *k*_on_ (L^3^T^-1^ in 3D, L^2^T^-1^ in 2D) (Huang et al., 2010). This difference in dimension increases the complexity of receptor-ligand interaction and makes it much more challenging to measure the receptor-ligand binding kinetics *in situ*. With the development of advanced technology, a series of approaches for measuring the 2D receptor-ligand binding kinetics, such as micropipette adhesion frequency and thermal fluctuation methods, have been developed and enable us to further investigate the 2D receptor-ligand interaction (Orsello et al., 2001; Williams et al., 2001). It has been confirmed that the 2D binding kinetics of receptors and ligands depend not only on their binding strength, but also on, e.g., external force, protein flexibility, and membrane fluctuation, in sharp contrast to the 3D case. Specifically, 2D measurements from the adhesion frequency and thermal fluctuation assays show that the T cell receptor (TCR) binds to peptide major histocompatibility complex (pMHC) with significantly more rapid on-rate and higher binding affinity, as compared with that measured in solution. The rapid kinetics and broad affinity of 2D TCR-pMHC interaction are proposed to determine the T-cell responsiveness. These results highlight the necessity and importance of studying 2D receptor-ligand interaction and stimulate the development of 2D measuring methods. In this Review, we summarize several mechanical-based methods, including micropipette adhesion frequency method (Chesla et al., 1998; Huang et al., 2010; Liu et al., 2014; Seo et al., 2016; Zhang et al., 2016), thermal fluctuation method (Evans et al., 1995; Chen et al., 2008a; Chen et al., 2008b), flow chamber method (Chesla et al., 1998), and fluorescence-based methods involving fluorescence recovery after photobleaching (FRAP) (Tolentino et al., 2008; Wu et al., 2008), and fluorescence resonance energy transfer (FRET) (Huppa et al., 2010). Some strengths and weaknesses of each method are briefly discussed.

## 2 Mechanical-based 2D methods

### 2.1 Micropipette adhesion frequency assay

**FIGURE 2:**
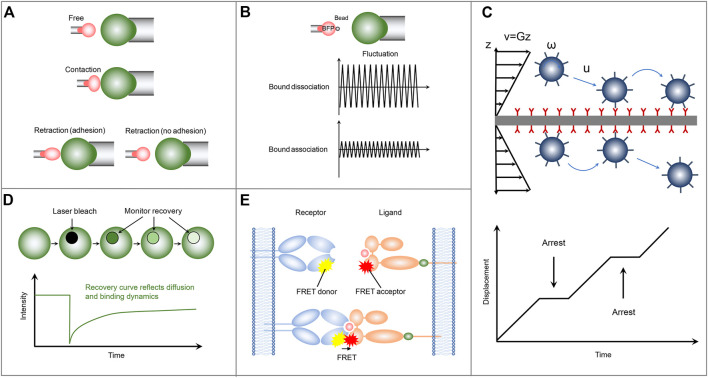
Schematics of the representative mechanical-based and fluorescence-based methods for measuring the two-dimensional receptor-ligand binding kinetics. **(A)** Micropipette adhesion frequency assay. **(B)** Thermal fluctuation method. **(C)** Flow chamber method. **(D)** FRAP. **(E)** FRET.

In the micropipette adhesion frequency assay, a micropipette-pressurized human red blood cell (RBC) is usually utilized to present ligands with a desired density, which can be determined by, e.g., flow cytometry analysis (Huang et al., 2010). Similarly, a nucleated cell with complementary receptors is also aspirated by another micropipette through applying an appropriate suck pressure and is placed to appose RBC (Figure 2A). The two apposing cells are then driven into contact with precisely controlled contact area and duration *via* micromanipulation. The adhesion events are identified by microscopically observing the deflection of the RBC membrane during the retraction of RBC away from the nucleated cell. Here, the RBC functions as an adhesion sensor due to its ultrasoft membrane, which can be deformed in response to subpiconewton force, an order of magnitude smaller than that required for breaking a receptor-ligand bond. Since the sensitivity of the measuring system, depending on the mechanical response of RBC to adhesion force, determines the accuracy of the experimental results, the sucking pressure regulated by the height of the reservoir becomes a key parameter. Repeating the contact-retraction cycle up to, e.g., one hundred times from several pairs of cells to determine the probability of cell-cell adhesion at that contact duration (Puech et al., 2011; Limozin et al., 2019). Based on a probabilistic model of small system kinetics, the 2D receptor-ligand kinetic information can be extracted by fitting the experimental data with the reaction kinetics equation, *P*_a_ = 1-exp{-*m*_r_ *m*_l_ *A*_c_ *K* [1-exp (-*k*_off_ *t*)]}, of the probability of adhesion (*P*_a_) with time (*t*). Where *m*_r_, *m*_l_ and *A*_c_ are the surface density of the receptor, the surface density of the ligand and the contact area respectively. The micropipette adhesion frequency assay with single-bond detection sensitivity and wide applicability has provided us with a wealth of 2D binding kinetics information of different receptor-ligand pairs. For example, using micropipette adhesion frequency method, Chesla et al. studied the interactions of Fcγ receptor IIIA (CD16A) with either human or rabbit immunogobulin G (IgG), which regulate various effector responses in the immune system, such as phagocytosis, cellular cytotoxicity, and antigen presentation (Chesla et al., 1998). Their results show that CD16A binds to rabbit IgG with a twofold faster on-rate, but with a twofold slower off-rate, as compared with that of human IgG, thus leading to a fourfold difference in their binding affinity.

### 2.2 Thermal fluctuation method

In the thermal fluctuation assay, a ligand-coated bead is attached to the apex of a micropipette-aspired RBC *via* specific receptor-ligand interaction and is aligned against a receptor-bearing cell aspirated by another micropipette (Evans et al., 1995) (Figure 2B). Partially due to its attachment to the ultrasoft RBC membrane, the bead undergoes thermal fluctuations, which can be suppressed by the formation of receptor-ligand bond at the interface between ligand-coated bead and receptor-functionalized cell. Monitoring precisely the target cell to a position at which the attached bead contacts with the target cell through its thermal fluctuation but not by compressing the target cell. Then by tracking accurately and rapidly the decrease and resumption of bead thermal fluctuations in real-time, the receptor-ligand bond formation and dissociation events, as well as the bond lifetime can be identified during the contact period. The receptor-ligand binding kinetic information including the on-rate and off-rate is then derived by fitting the waiting time distribution and bond lifetime distribution according to a first-order kinetic model (Huppa et al., 2010). In the micropipette adhesion frequency assay, only the information of whether the adhesion occurs at the end of the contact time is required. In contrast, comprehensive information, including the receptor-ligand bond formation and dissociation events, as well as the bond lifetime at a single-bond level, can be obtained in the thermal fluctuation assay with higher temporal and spatial resolution (Chen et al., 2008b), thus improving the reliability and robustness of the measured receptor-ligand binding kinetics. Using the thermal fluctuation method, Chen et al. (2008a) performed a kinetic experiment to investigate the interaction of P-selectin glycoprotein ligand-1 (PSGL-1) with L-selectin or P-selectin. They found that PSGL-1 has a slower on-rate, but a faster off-rate, with L-selectin than P-selectin, providing insight into the leukocyte rolling adhesion on the surfaces of vascular endothelial cells during inflammation.

### 2.3 Flow chamber method

Another representative mechanical-based 2D method is the flow chamber assay, in which receptor-bearing cells or receptor-encapsulated microparticles are drawn into a chamber floor functionalized with cognate ligand molecules. A pump-controlled laminar fluid flow with varied velocity is then introduced to subject the cells or microparticles to shear stress (Figure 2C). These cells or microparticles undergo adhesion, detachment, and reattachment to the surface of the chamber floor in the upright orientation during their rolling process in response to the laminar fluid flow. By monitoring the position and trajectory of rolling cells or microparticles with a computer-based tracking system, two independent adhesion information, i.e., binding frequency and detachment kinetics, can be then obtained, from which the receptor-ligand off-rate can be determined by fitting the adhesion curve of the number of events remaining bound as a function of time. Compared with the adhesion frequency and thermal fluctuation assays, the flow chamber assay has higher throughput and can be used to investigate the response of the binding kinetics to the external force by applying a range of shear stresses (Robert et al., 2012). Note that, the flow chamber assay with chamber floor in the upright orientation is unable to measure the on-rate and the binding affinity. To obtain more binding kinetic information, a minimal mathematical rolling model is proposed for a flow chamber assay with the chamber floor in the inverted orientation. Using the inverted flow chamber assay, Li et al. (2016) evaluated the on-rate and binding affinity of selectin-ligand interactions, which are found to increase in response to enhanced shear stresses within a certain range. Their results provide insights into the molecular mechanism underlying the flow-enhanced stability of rolling leukocytes.

## 3 Fluorescence-based 2D methods

### 3.1 Fluorescence recovery after photobleaching (FRAP)

The FRAP assay consists of a receptor-bearing cell adhering on a supported lipid bilayer (SLB) functionalized with cognate ligands, which can diffuse freely across the SLB and are tagged with fluorescent labels to track their diffusion and quantify their molecular densities. Upon the cell adhesion on the SLB, receptors start to bind ligands. The formation of receptor-ligand complex significantly reduces the number of free ligands at the contact area, which leads to the free ligands diffusion from outside to inside the contact area and the accompanying accumulation of fluorescence in the contact area as a result of the density gradient. The binding affinity of receptor and ligand can be derived by analyzing the bound and free ligand density at equilibrium in the contact area according to Golan-Zhu plot (Zhu et al., 2007) or Scatchard plot (Dustin et al., 1996). To further explore the receptor-ligand association/dissociation kinetics, FRAP-based method is adopted in the cell-SLB adhesion system. Specifically, the ligand fluorescence in contact area is bleached with a laser pulse. Due to the receptor-ligand dissociation, rebinding, and the exchange of bleached ligands for fluorescent ligands by diffusion, the fluorescence recoveries over time in the contact area (Figure 2D). Using a diffusion-reaction model (Wu et al., 2008), one can evaluate the on-rate and off-rate by fitting the fluorescence recovery curve, which provides information on ligand diffusion and receptor-ligand binding kinetics. FRAP-based assay provides a new method for measuring the 2D receptor-ligand kinetic rates and can be used to simultaneously investigate the retarded diffusion and non-recoverable fractions of interacting proteins. But not all fluorescent proteins are irreversibly photobleached, and this behavior can lead to false results. Using this FRAP assay, Tolentino et al. (2008) found that the 2D off-rate is at least two orders of magnitude slower than that measured in 3D solution and observed a significant non-recoverable fraction of interaction ligands, which might be important for the formation of stable signaling platforms.

### 3.2 Fluorescence resonance energy transfer (FRET)

Another fluorescence-based protocol for measuring the receptor-ligand binding kinetics is the single-molecule FRET (Figure 2E). In contrast to FRAP, in which only the ligands on the SLB are labeled with fluorescence, in the single-molecule FRET method both receptor and ligand are respectively conjugated with donor and acceptor fluorophores. The receptor-ligand interaction brings the donor and acceptor fluorophores closer to each other to enable FRET. The association and dissociation kinetics and lifetime of receptor-ligand complex can be directly identified by monitoring the occurrence and disappearance of FRET signal. Using a single-step dissociation model (Huppa et al., 2010), the 2D receptor-ligand off-rate can be determined by fitting the FRET disappearance events as a function of time. And the 2D receptor-ligand affinity is calculated by analyzing the area concentrations of receptor-ligand complex, free receptor, and free ligand, which are estimated from the donor/acceptor fluorescent intensities and the FRET signals. Using this FRET assay, Huppa et al. investigated the binding kinetics of TCR and pMHC. They found an approximately 100-fold and 4-12-fold increase in the 2D affinity and off-rate, respectively, compared with the 3D results measured by SPR. Their results indicate that the elevated 2D TCR-pMHC affinity results from their fast on-rate (Huppa et al., 2010). This elegant FRET protocol enables us to investigate the 2D receptor-ligand interactions at the single molecule level (Huang et al., 2012). However, the unique FRET design and generation required for each molecular system lead to high technical challenges. Moreover, to sensitively capture the weak single-molecule FRET signal to ensure the accuracy of measured results, the single-molecule FRET assay should also be equipped with a fine-tuned optical system (Zhu et al., 2013).

## 4 Discussion

Here, we summarize several mechanical-based and fluorescence-based methods developed for measuring the 2D receptor-ligand binding kinetics. Some strengths and weaknesses of each method are briefly discussed. The experimental results from these measuring methods have further enriched our understanding of the 2D receptor-ligand interactions and revealed a sharp contrast between the measured data from 2D biologically inspired approach and 3D reductionist approach, which in turn prompts a rethinking of our current views of the *in situ* receptor-ligand interactions.

It will further contribute to our understanding on the receptor-ligand interaction through a combination of experimental and computational methods. For example, theoretical and computer simulation results reveal the thermal membrane roughness-induced binding cooperativity of 2D receptor-ligand interaction (Krobath et al., 2009; Hu et al., 2013). This binding cooperativity, which has been subsequently confirmed in the CD47-SIRPα mediated adhesion system (Steinkühler et al., 2018), helps to explain the several orders of magnitude difference in the binding affinity measured by different experimental methods. Moreover, experimental results from the adhesion frequency and thermal fluctuation assays using a micropipette indicate that disrupting the membrane microdomains, often termed lipid rafts, with methyl-β-cyclodextrin reduces the binding affinity of TCR and pMHC (Huang et al., 2010). To illuminate the mechanism underlying the raft-regulated receptor-ligand interaction, Li et al. developed a multicomponent membrane adhesion system in the framework of classical statistical mechanics and systematically studied the interplay between the receptor-ligand interaction and lipid raft (Li et al., 2017a; Li et al., 2017b; Li et al., 2018; Li et al., 2020; Li et al., 2021d; Li et al., 2021e; Li et al., 2021c; Li et al., 2022; Li et al., 2023). They found that lipid rafts enhance the receptor-ligand binding affinity, consistent with the experimental results, and the receptor-ligand binding in turn contributes to the raft coalescence. The membrane fluctuation and the induced entropic force are revealed to play a central role. Their findings uncover the novel mechanism and significantly deepen our understanding on the physiological function of both receptor-ligand interaction and lipid raft. In addition, their further studies using the developed statistical mechanical model show that the entropic force-mediated raft coalescence is also implicated in the pattern formation and receptor-ligand binding during T-cell adhesion, which helps to explain recent experimental observations and provides potential new therapeutic options for immunotherapy (Li et al., 2021b). These results together embody fully the great importance of the combination of experimental and computational methods in gaining insight into the receptor-ligand binding, and further studies should focus on the synergistic development of experimental and computational methods.
